# Diuretic Use in Heart Failure

**DOI:** 10.31083/RCM39547

**Published:** 2025-10-20

**Authors:** George Nassar, Robert Jameson

**Affiliations:** ^1^Department of Internal Medicine, Houston Methodist Hospital and Weill Cornell Medicine, Houston, TX 77030, USA; ^2^Nephrology, Dialysis and Transplantation Associates, Houston, TX 77030, USA

**Keywords:** diuretics, heart failure, volume status, congestion, diuretic resistance

## Abstract

Heart failure (HF) is a major contributor to morbidity and mortality in the US and worldwide. HF is a complex condition characterized by the disruption of normal physiology and the activation of neurohumoral pathways, including the renin–angiotensin–aldosterone system, the sympathetic system, and inflammatory pathways. These have adverse effects on renal handling of salt and water balance, leading to salt and water retention and a vicious cycle of worsening congestive changes with progressive volume overload. Meanwhile, diuretics are pharmacologic agents that are essential in the management of HF. Indeed, diuretics induce natriuresis to disrupt this vicious cycle of progressive volume overload, thereby reducing congestive changes and alleviating the symptoms of HF. In this review, we discuss the different classes of diuretics and their sites and mechanisms of action across the nephron. We highlight differences in the potency and usefulness of these diuretics. Moreover, we examine their application in the management of various stages of HF, focusing on their optimal and effective use in clinical practice. In this review, we also cover several aspects of the pathophysiology of HF. We address the milder forms of HF that are treated in outpatient clinics, as well as the more advanced states of HF, including acute decompensated HF (ADHF), which are usually managed in a hospital setting. We discuss management strategies in the outpatient setting, with a specific focus on maintaining sufficient decongestion of patients to prevent hospitalization. We stress the importance of closely monitoring congestive symptoms and weight trends, as well as electrolyte and renal parameters. We recommend setting a “target weight goal” for the patient between clinic visits, which helps with outpatient diuretic therapy adjustments and avoids drifts in volume status. We also examine the usefulness of diuretics in hospitalized patients with ADHF. For these more challenging conditions, we discuss the use of combination diuretics to overcome diuretic resistance and highlight current recommendations for achieving the desired goals and speed of diuresis. Patients with HF commonly have chronic kidney disease (CKD), which frequently complicates overall management strategies. CKD also leads to diuretic resistance, necessitating escalation of diuretic dosing and more frequent changes in diuretic prescription. Hence, this review also discusses management strategies for CKD patients and highlights the importance of close monitoring of kidney function in both inpatient and outpatient settings when using diuretics in patients with HF. We briefly discuss the benefits of monitoring central venous filling pressures in patients with ADHF as a tool to guide the optimization of diuresis. Finally, we allude to new advanced technologies such as remote monitoring of outpatients with HF. These can be used to detect early signs of impending HF decompensation that earlier adjustments to the diuretic dose could then address.

## 1. Introduction

Heart failure (HF) is a major global health challenge, affecting over 64 million people worldwide and contributing significantly to morbidity, mortality, and healthcare expenditures [1, 2, 3, 4]. Recent epidemiologic studies continue to document an increase in HF prevalence, particularly among aging populations and individuals with comorbid conditions such as hypertension, diabetes, and chronic kidney disease (CKD) [5]. Regardless of whether the etiology is ischemic, hypertensive, valvular, or idiopathic, HF ultimately results in the heart’s inability to maintain adequate cardiac output. In response, neurohormonal pathways including the renin-angiotensin-aldosterone system (RAAS), the sympathetic nervous system, and natriuretic peptides are activated to maintain circulatory homeostasis. However, these compensatory mechanisms often become maladaptive, promoting sodium and water retention, increasing preload, and worsening myocardial function, thus perpetuating a cycle of volume overload and decompensation [6].

Diuretics are essential pharmacologic agents that interrupt the above pathophysiologic cycle by inducing natriuresis and diuresis [7]. Although they do not demonstrate a reduction in mortality, their value lies in the rapid alleviation of congestive symptoms such as dyspnea, peripheral edema, and elevated jugular venous pressure [8]. Both the American (American College of Cardiology (ACC)/American Heart Association (AHA)/Heart Failure Society of America (HFSA)) and European Society of Cardiology (ESC) HF guidelines recommend loop diuretics as first-line agents for congestion management, with an emphasis on individualized dosing based on clinical volume status and renal function [9, 10]. These recommendations affirm the central role of diuretics in symptom relief across the spectrum of HF severity. Diuretics are thus the cornerstone of symptom-directed therapy in HF, particularly during episodes of acute decompensated heart failure (ADHF), where prompt decongestion is critical to stabilize hemodynamics and relieve end-organ stress [8].

This review will discuss the use of diuretics in the management of HF. We first provide a brief overview of the different pharmacologic classes of diuretics, their nephron-specific sites, and mechanisms of action. We then examine their use across the HF spectrum, ranging from stable outpatient management to hospital-based care of ADHF. We discuss challenges imposed by diuretic resistance, often driven by coexisting CKD or neurohormonal activation, which can significantly hinder effective volume management. To overcome such diuretic resistance, we explore diuretic escalation strategies including the application of sequential nephron blockade with combination diuretic therapy to achieve diuresis and decongestion goals [9].

The effective and safe use of diuretics demands close monitoring. In the hospital setting, real-time feedback provided by daily weights, urine volume, urinary sodium concentration, and central venous pressure trends is used to guide diuresis intensity. In outpatient care, the monitoring of serum creatinine, electrolytes, and weight trends is essential to protect against kidney failure and electrolyte derangements, provide guidance for dose adjustments, and prevent readmissions [9]. We recommend establishing an individualized “target weight” as a reference point to guide outpatient therapy, promote euvolemia, and minimize the risk of recurrent decompensation.

## 2. Diuretics: General Classes and Differences

### 2.1 Classes of Diuretics

**Fig. 1.**
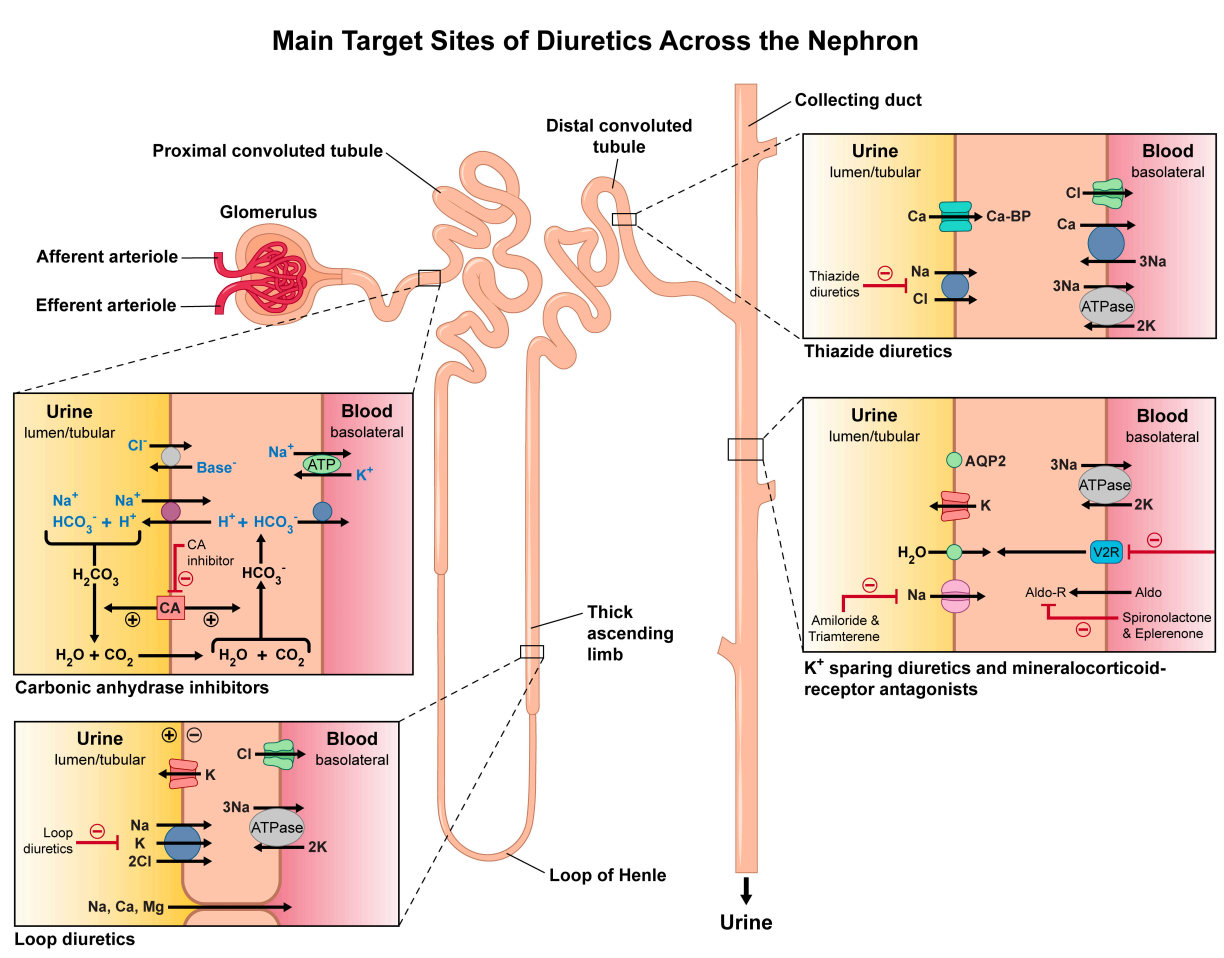
**Main target sites of diuretics across the nephron**. Illustrated here are the nephron segments targeted by different diuretic agents. Carbonic anhydrase inhibitors act on the proximal convoluted tubule. Loop diuretics target the thick ascending limb of the loop of Henle. Thiazide diuretics act on the distal convoluted tubule. Potassium-sparing diuretics and mineralocorticoid receptor antagonists act within the collecting duct. The diagram also highlights the transport pathways and key ion exchanges modulated by each class to promote natriuresis and diuresis.

Historically, the term “diuretics” refers to natriuretic diuretics. However, with the advent of aquaretic diuretics [11], it is important to distinguish between the two mechanisms of diuresis. Natriuretic diuretics act to reduce sodium absorption at specific sites in the renal tubule (Fig. 1), thereby increasing sodium and water excretion. The ability to enhance urinary sodium excretion is essential for the management of HF and edematous states. The main classes of diuretics are summarized in Table 1. In contrast, aquaretic diuretics mediate their effects by blocking vasopressin receptors to promote water excretion without significantly altering sodium excretion. The role of aquaretic diuretics in the management of HF is largely to manage hyponatremia. For the remainder of this review, the term diuretic will refer to natriuretic diuretics.

**Table 1.** **Review of diuretic types, mechanisms and uses**.

Class	Example drugs	Site of action	Mechanism of action	Common uses
Loop Diuretics	Furosemide	Thick ascending limb of the loop of Henle	Inhibit	Edema
	Bumetanide		Na/+K^+^-ATPase	hypertension
	Torsemide		cotransporter	
	Ethacrynic acid			
Thiazide Diuretics	Hydrochlorothiazide	Distal convoluted tubule	Inhibit	Hypertension
	Chlorthalidone		Na^+^-Cl^−^	mild edema
	Indapamide		symporter	calcium nephrolithiasis
	Metolazone			osteoporosis
Potassium-Sparing Diuretics	Amiloride	Late distal tubule and collecting duct	Inhibit epithelial sodium channels (ENaC)	Diuretic associated hypokalemia
	Triamterene		reducing sodium reabsorption and potassium excretion	Diuretic augmentation
Aldosterone Antagonists	Spironolactone	Aldosterone receptors of the collecting duct	Block aldosterone receptor, reducing sodium reabsorption and potassium excretion	Heart failure
	Eplerenone			Primary hyperaldosteronism resistant hypertension
Carbonic Anhydrase Inhibitors	Acetazolamide	Proximal convoluted tubule	Inhibit carbonic anhydrase, reducing bicarbonate reabsorption	Glaucoma
	Methazolamide			altitude sickness
				metabolic alkalosis
Osmotic Diuretics	Mannitol	Proximal tubule and descending limb of the loop of Henle	Increase osmolarity of filtrate, reducing water reabsorption	Intracranial or intraocular pressure reduction

Classes of diuretics used in clinical care, with representative drugs, sites and mechanisms of action, and common clinical applications such as heart failure (HF), hypertension, volume overload, and electrolyte disturbances.

### 2.2 Classes of Diuretics in Clinical Care

Thiazide diuretics, such as hydrochlorothiazide (HCTZ), act primarily on the distal convoluted tubule to inhibit the sodium-chloride (Na^+^-Cl^–^) cotransporter, which is responsible for absorption of a small percentage (3–5%) of filtered sodium [12] (Fig. 1). This modest reduction in tubular sodium reabsorption leads to moderate diuresis. Thiazide diuretics are frequently used as primary or supplementary agents in treating hypertension. Chlorthalidone is a longer acting thiazide diuretic than HCTZ and is more effective as once daily dosing [12, 13]. Thiazides may be used successfully for the chronic management of milder forms of HF. However, their efficacy is significantly reduced in CKD states, and they are unlikely to be effective as the sole diuretic agents in more advanced states of HF.

Loop diuretics, such as furosemide, act on the ascending limb of the loop of Henle, where they inhibit the sodium-potassium-chloride (Na^+^-K^+^-2Cl^–^) co-transporter [5] (Fig. 1). This action results in a potent natriuretic effect, leading to the excretion of 20–25% of filtered sodium. Loop diuretics are fast-acting, making them highly effective in acute and critical settings such as pulmonary edema or severe volume overload. However, their efficacy is reduced in hypoalbuminemia and moderate to severe CKD, and higher and more frequent dosing regimens may be required in these situations to overcome diuretic resistance. Torsemide and bumetanide are more potent loop diuretics and have better absorption kinetics than furosemide, thus enhancing their utility in managing diuretic resistance. The pharmacologic characteristics of commonly used loop diuretics are compared in Table 2 and will be further explored in this review [5].

**Table 2.** **Pharmacologic characteristics of commonly used loop diuretics**.

Drug	Bioavailability	Bioavailability in edematous states	Onset (min)	Half-Life (h)	Duration (h)	Potency vs. Furosemide	Clinical notes
Furosemide	50%	10–60%	30–60	1.5	6–8	1x	Most used; variable absorption makes dosing less predictable in HF and cirrhosis.
Bumetanide	80–100%	Preserved	30–60	1.0	4–6	20–40x	Used when more predictable effect is needed.
Torsemide	80–100%	Preserved	30–60	3.5	6–8	2–4x	Used when more predictable effect is needed; well-studied in chronic HF.
Ethacrynic Acid	100%	Preserved	30	1.5	6–8	0.8x	Sulfa-free alternative; more ototoxicity risk compared to others.

This table summarizes the key properties of orally administered loop diuretics, including bioavailability in general and in edematous states, potency, onset and duration of action, half-life, and common clinical uses. Notably, furosemide shows highly variable and often reduced absorption in edematous patients, whereas bumetanide and torsemide have more reliable bioavailability, which may influence diuretic selection in HF and other volume-overloaded conditions.

Potassium-sparing diuretics act in the principal cells of the collecting tubules, as shown in Fig. 1. Sodium reabsorption in these segments occurs through mineralocorticoid-sensitive sodium channels and is associated with secretion of potassium and hydrogen ions [5]. Amiloride and triamterene exert their potassium-sparing diuretic effects through direct blockade of these sodium channels, whereas spironolactone, eplerenone and finerenone exert their effects through competitive inhibition of the mineralocorticoid receptor [5]. Although potassium-sparing diuretics have weak natriuretic effects, their inhibition of sodium absorption by the principal cells makes them useful agents in reducing potassium losses induced by other classes of diuretics. Their use in HF extends beyond just their diuretic effects, as they antagonize the unwanted effects of hyperaldosteronism on the heart and are associated with improved outcomes in HF [5, 14].

Carbonic anhydrase inhibitors, such as acetazolamide, act on the proximal tubules by inhibiting carbonic anhydrase [14, 15] (Fig. 1). This action reduces bicarbonate reabsorption and promotes sodium and water excretion. However, their net overall diuretic efficacy is relatively weak because most of the sodium and water that escapes reabsorption in the proximal tubule is subsequently reclaimed in the loop of Henle. Despite their limited stand-alone diuretic effect, carbonic anhydrase inhibitors play a critical role in specific clinical scenarios. They are often used in combination with other diuretics in HF management to correct metabolic alkalosis, a common side effect of the prolonged use of other diuretic classes.

Osmotic diuretics such as mannitol are not used in the management of HF due to concerns about their mechanism of action. These agents increase osmotic pressure within the renal tubules, drawing water into the urine independently of sodium excretion. However, their hypertonic properties can lead to initial expansion of intravascular volume, which may exacerbate congestion in patients with HF and worsen hemodynamic status [16].

### 2.3 Differences Between Loop Diuretics

Furosemide, bumetanide, and torsemide are three commonly used loop diuretics with distinct differences in their absorption profiles, pharmacokinetics, and potency (Table 2) [6, 17]. Furosemide is the most widely used worldwide, but its oral bioavailability is highly variable, ranging from 10–90%. Moreover, its bioavailability is significantly reduced in patients with edematous gut conditions, such as HF or cirrhosis, leading to inconsistent diuretic effects in these settings. Torsemide has a more consistent bioavailability of 80–100%, and its absorption is much less affected by edematous states compared to furosemide. Additionally, torsemide has a longer half-life compared to furosemide, which can result in more sustained diuretic effects. Bumetanide also has more reliable oral absorption than furosemide, with bioavailability consistently around 80–90%. Both torsemide and bumetanide are more predictable choices for patients with compromised gut absorption.

In terms of potency, torsemide is more potent than furosemide, with 10–20 mg of torsemide equating to 40 mg of furosemide. Bumetanide is also more potent than furosemide, with approximately 1 mg of bumetanide being equivalent to 20–40 mg of furosemide. This increased potency can be advantageous in managing patients who require higher oral doses of loop diuretics. Hence, the choice for oral administration among these loop diuretics should consider the patient’s specific clinical situation, including their gut absorption capacity, diuretic resistance, and the required dose and potency to achieve the desired diuretic response [6]. When administered intravenously, the differences in potency among these loop diuretics are maintained. However, their maximal diuretic effect is similar when given in bioequivalent doses. Hence, their overall maximal effect on diuresis and sodium excretion is comparable when adjusted for dose. This similarity in maximal intravenous (IV) effect allows clinicians to use the drugs interchangeably in acute settings that demand IV administration, with the understanding that dose adjustments will equalize their efficacy.

### 2.4 Differences Between Thiazide Diuretics

Thiazide diuretics vary in potency, half-life, and effectiveness [13, 14, 15]. HCTZ is the most widely used and has moderate potency and a half-life of 6–15 hours, allowing for once or twice daily dosing. Chlorthalidone is more potent than HCTZ and has a significantly longer half-life of 40–60 hours, which allows for once-daily dosing and provides a more sustained anti-hypertensive effect. This extended half-life increases its effectiveness in managing fluid overload in congestive HF (CHF). Chlorothiazide is available in both oral and IV forms and is useful in acute settings. Its potency is comparable to HCTZ, but with a shorter half-life of about 90 to 120 minutes when given intravenously, making it suitable for eliciting a rapid diuretic response in hospitalized patients.

All thiazide diuretics share a common mechanism of action, which is the inhibition of sodium reabsorption in the distal convoluted tubule [14]. The choice of an appropriate thiazide diuretic in CHF management depends on the clinical context, the required potency, and the desired duration of action, thus balancing efficacy with the risk of electrolyte imbalances. While thiazides can reduce blood pressure and edema, they are less effective diuretic agents in advanced CKD and severe CHF patients.

## 3. Heart Failure: Scope, Classification and Pathophysiology

### 3.1 Scope of HF in the United States and Worldwide

HF poses a growing threat to global health, affecting more than 64 million individuals worldwide and placing a substantial burden on healthcare systems through increased morbidity, mortality, and economic costs [1, 2]. Recent global assessments highlight the persistent increase in prevalence of HF across diverse populations [3, 4]. In the United States, HF affects approximately 6 million adults, with projections this will exceed 8 million by 2030 [1, 18]. The annual incidence is around 1 million new cases. Epidemiological data show a steady increase in HF incidence, particularly in older adults and those with underlying comorbidities including hypertension, diabetes mellitus, and CKD [5]. HF is associated with a five-year mortality rate of almost 50%. It results from structural and/or functional cardiac abnormalities, and presents with signs of systemic and pulmonary congestion and elevated brain natriuretic peptide (BNP) levels. While a comprehensive discussion of HF is beyond the scope of this review, the following section provides a concise overview so that subsequent therapeutic considerations can be contextualized.

### 3.2 Heart Failure Classification

The classification of HF is crucial for managing this condition. The New York Heart Association (NYHA) functional classification [9] is one of the most widely used systems and is the most relevant for the scope of the present review [9]. It categorizes HF into four classes based on the severity of symptoms and physical limitations. Class I includes patients with no limitation of physical activity: ordinary physical activity does not cause undue fatigue, palpitations, or shortness of breath. Class II involves slight limitation of physical activity: patients are comfortable at rest, but ordinary activity results in symptoms. Class III denotes marked limitation of physical activity: patients are comfortable at rest, but less than ordinary activity causes symptoms. Class IV represents patients who are unable to carry out any physical activity without discomfort, and may have symptoms even at rest.

The ACC and the AHA offer another classification system that is focused on the different stages of HF development [9]. Stage A includes individuals who have an elevated risk for HF, but without structural heart disease or symptoms. Stage B includes patients with structural heart disease, but no symptoms of HF. Stage C includes patients with structural heart disease who also have current or past symptoms of HF. Stage D includes patients with refractory HF requiring specialized interventions.

The two classification systems are complementary, with the NYHA functional classification providing insight into the patient’s current physical capabilities and symptom severity, while the ACC/AHA system emphasizes the progression and underlying structural changes of HF. These classification systems help to predict outcomes for HF patients and guide treatment decisions. Diuretic use is just one component of the multifaceted and multidisciplinary approaches required to manage HF patients. Lifestyle modifications and dietary changes are also critical components of care in these patients.

### 3.3 Left Heart Failure 

The most common type of HF is left-sided HF, which occurs when the left ventricle (LV) is unable to pump blood efficiently throughout the body. This is the most prevalent and recognizable form of HF. Understanding the differences between various forms of left HF is crucial for accurate diagnosis and treatment [18].

Left HF often results from conditions such as coronary artery disease, hypertension, and cardiomyopathy. When left HF is associated with reduced LV ejection fraction (LVEF) as a result of weakened heart muscle, it is named HF with reduced ejection fraction (HFrEF). When left HF is associated with preserved LVEF, it is named HF with preserved ejection fraction (HFpEF). The latter occurs due to stiffened heart muscle that impairs LV relaxation and filling. Both states of left HF lead to reduced cardiac output and increased pressure in the left atrium and pulmonary artery. This in turn induces pulmonary congestion and edema. Symptomatology includes dyspnea, orthopnea, paroxysmal nocturnal dyspnea, and fatigue. Patients usually experience exercise intolerance and fluid retention, leading to pulmonary rales and peripheral edema. Effective management includes addressing the underlying cause with disease-modifying medications such as angiotensin converting enzyme inhibitors (ACE inhibitors), angiotensin receptor blockers (ARBs), beta-blockers, aldosterone blockers, and sodium glucose transporter 2 inhibitors (SGLT2i). These medications constitute guideline-directed medical therapy (GDMT). In addition, diuretics are often necessary to optimize fluid balance, reduce pulmonary congestion, and alleviate clinical symptoms.

### 3.4 Pulmonary Hypertension

Pulmonary hypertension is characterized by elevated luminal pressure in the pulmonary arteries, often due to acquired or idiopathic chronic lung diseases, left HF, or pulmonary thromboembolism [19]. Pathophysiologically, increased resistance in the pulmonary vasculature leads to right ventricular hypertrophy and eventual failure. Symptoms include dyspnea, fatigue, chest pain, syncope, fluid retention, abdominal distention, and lower extremity edema. Advanced cases may present with massive fluid retention and compromised renal function. Management includes addressing the underlying cause of pulmonary hypertension, and the use of pharmacologic agents to decrease pulmonary vascular resistance. Diuretics are used to manage fluid retention and reduce right ventricular congestion, peripheral edema, and ascites. Prostacyclin analogs, endothelin receptor antagonists, and phosphodiesterase-5 inhibitors are commonly used to treat primary pulmonary arterial hypertension.

### 3.5 Right Heart Failure

Right HF often results from conditions such as right ventricular infarction, valvular heart disease, chronic lung diseases (e.g., COPD and interstitial lung disease), pulmonary arterial hypertension, or left HF. It occurs when the right ventricle fails to pump blood efficiently to the lungs [19]. Symptoms include peripheral edema, ascites, hepatomegaly, and jugular venous distention. Patients may also experience fatigue and abdominal discomfort. Management includes addressing the underlying cause, and the use of medications such as RAAS inhibitors, beta-blockers, SGLT2i, and diuretics to improve heart function. The proper management of fluid balance by using diuretics is crucial for alleviating symptoms and reducing complications.

### 3.6 Other Forms of Heart Failure

Other forms of HF can still include fluid retention, such as valvular heart disease, congenital heart disease, and high output HF. Treating the underlying anatomic derangements associated with these conditions is the most important therapeutic measure. Diuretics are also relevant in these conditions, but should be incorporated into a comprehensive treatment plan unique to each individual case.

### 3.7 Neurohumoral Activation in Advanced HF

The pathophysiology of advanced HF and ADHF, includes both arterial underfilling and venous congestion. Together, these derangements lead to activation of the RAAS pathway, sympathetic nervous system, and inflammatory pathways. This results in increased renal arterial vasoconstriction, decreased glomerular filtration rate (GFR), retention of salt and water, increases in central venous pressure (CVP) and pulmonary capillary wedge pressure (PCWP), and the emergence of symptomatic edema and volume overload. As these pathophysiologic and neurohormonal derangements become more advanced in ADHF, they lead to diuretic resistance [20, 21].

## 4. Heart Failure With Kidney Disease

### 4.1 Prevalence

Kidney failure is highly prevalent in HF patients, with up to 50% suffering from CKD [2]. The causes of kidney failure may be (1) co-existing CKD, (2) progressive kidney disease due to chronic HF, (3) a precipitous decline in kidney function due to the development of acute or subacute decompensated cardiorenal syndrome (CRS), or (4) acute tubulointerstitial kidney injury unrelated to HF.

### 4.2 Management Challenges

There are several ways in which kidney disease complicates the management of HF patients. (1) It increases the tendency for fluid overload and hypertension. (2) It creates diuretic resistance. (3) It may limit the use of GDMT pharmaceutical agents which could adversely affect kidney function. (4) It worsens patient outcomes, leading to higher morbidity, mortality, and hospitalization rates.

The interplay between HF and kidney failure creates a vicious cycle, where the worsening of one condition leads to deterioration of the other [22]. ADHF leads to reduced kidney perfusion and worsening kidney function. Conversely, worsening kidney disease can exacerbate HF by increasing fluid retention, exacerbating hypertension, and causing diuretic resistance. Furthermore, the diuretics commonly used in HF can lead to electrolyte imbalances and additional renal impairment. These adverse effects necessitate a multidisciplinary approach to management, involving cardiologists, nephrologists, and primary care providers. Regular monitoring of kidney function is essential to allow appropriate adjustment of treatment plans. Patients with both HF and CKD often require more frequent medical visits and more intensive care. Diuretic use in CKD patients is more challenging due to diuretic resistance. It requires additional expertise in diuretic management because of the need to use escalating doses and a combination of diuretics. Moreover, the presence of baseline CKD means there is a greater tendency for more abrupt fluctuations in renal function, thus requiring more frequent adjustment of the diuretic management strategy.

## 5. Use of Diuretics for Stable HF Patients in the Outpatient Setting

### 5.1 Physiological Rationale for Diuretic Use in HF

While diuretics serve multiple clinical purposes, including the treatment of hypertension, edema, and disturbances in acid-base and electrolyte balance, this review will focus on their role in HF. Diuretics are central in the symptomatic management of HF, primarily by addressing fluid overload. Diuretics inhibit sodium and chloride reabsorption at various sites in the nephron. Of relevance is their inhibition of sodium absorption at the loop of Henle and the distal tubule. This inhibition promotes effective natriuresis and diuresis. As a result, intravascular volume decreases, which in turn decreases ventricular preload and PCWP. The ensuing decongestion alleviates common HF symptoms such as dyspnea, peripheral edema, and orthopnea. From a physiological standpoint, this decongestion improves ventricular compliance and reduces neurohormonal activation of the RAAS and sympathetic nervous system, both of which are drivers of HF progression. Although diuretics do not improve long-term survival in HF, their ability to correct volume overload and restore water-salt homeostasis significantly improves patient quality of life and functional status. Therefore, diuretics remain a key element of evidence-based HF management [9, 10].

### 5.2 Guideline-based Use of Diuretics in HF

The joint ACC, AHA, and HFSA guidelines from 2022 strongly recommend loop diuretics as first-line agents for relieving symptoms of congestion in patients with HF, particularly those with HFrEF [9]. The guidelines recommend individualized dosing based on clinical volume status, renal function, and symptomatic response, with close monitoring of electrolytes, weight, and kidney function. Clinicians are advised to carefully titrate diuretics to achieve euvolemia, while avoiding both under- and over-diuresis. Similarly, the 2021 European Society of Cardiology (ESC) guidelines recommend the use of loop diuretics to alleviate signs and symptoms of fluid retention across the spectrum of HF phenotypes, including HFrEF, HF with mildly reduced EF (HFmrEF), and HFpEF [10]. They recommend starting at the lowest effective dose and then adjusting upward based on the response. Both sets of guidelines acknowledge the challenge of diuretic resistance and recommend strategies such as sequential nephron blockade (e.g., adding thiazides) and IV diuretic administration when needed, particularly in cases of acute ADHF. Although diuretics do not provide a benefit in terms of reduced mortality, both the American and European cardiology societies emphasize the central role of diuretics in managing volume overload and improving the functional status and quality of life of HF patients. Both guidelines emphasize the fact that diuretics are a vital component of comprehensive GDMT and should be used in conjunction with disease-modifying agents.

### 5.3 General Approaches to Diuretic Choices in Outpatient Clinics

Diuretics play a useful role in the management of milder and compensated forms of HF in the outpatient clinic, where they alleviate symptoms by maintaining euvolemia. In addition, diuretics help to reduce pulmonary congestion and improve exercise tolerance.

Although the guidelines recommend loop diuretics for managing HF, thiazide diuretics may be initiated and used as sole agents for milder forms of HF in clinical practice. The typical approach to using thiazide diuretics involves starting with HCTZ and reserving chlorthalidone as a second-line option if a stronger diuretic effect is needed. Chlorthalidone is a more reliable choice due to its longer half-life and more sustained diuretic action. In practice, either of these thiazide diuretics can be effective as a first-line treatment. However, if the natriuretic response is suboptimal, loop diuretics could be added as part of a structured combination diuretic regimen, or they could be used initially as needed for breakthrough symptoms or signs of worsening fluid retention. If the HF advances and symptoms worsen, loop diuretics could then be used as the main or sole diuretic agent. Also, if the patient has significant CKD (GFR <30 mL/min/1.73 m^2^), it would be best to initiate diuresis with loop diuretics and avoid the use of thiazides altogether.

Furosemide is typically the first-line agent in the sequence of loop diuretic use. Treatment often begins with a 20 mg dose once or twice daily, with gradual escalation up to 80 mg two to three times daily as a near-maximum dose. Further increases beyond this dose do not usually provide additional benefit. Therefore, if such high doses are reached or prove ineffective, it is recommended to switch to torsemide or bumetanide due to their superior enteric absorption. When transitioning to torsemide, the initial oral dose should be approximately 50% of the most recent furosemide dose. For bumetanide, the starting oral dose should be around 1 mg for every 20 mg of oral furosemide.

When using combination diuretics, a loop diuretic can be paired with a thiazide diuretic to enhance natriuresis. For example, a loop diuretic may be added to a stable dose of chlorthalidone or hydrochlorothiazide. However, when loop diuretics form the primary regimen, metolazone is often the preferred add-on diuretic agent to boost natriuresis. Metolazone is a thiazide-like diuretic that is well absorbed and has a long half-life, making it particularly effective in overcoming diuretic resistance, especially in patients with CKD. It works by preventing the distal nephron from compensating for the natriuresis induced by the action of the loop diuretic in the thick ascending limb of the loop of Henle.

Metolazone is typically used on an as-needed basis. Effective doses can be as low as 2.5 mg daily, although higher doses of up to 10 mg per day may be required in some cases. However, adding metolazone to a stable loop diuretic regimen can occasionally cause excessive diuresis. Consequently, it is not usually administered daily but rather as needed, or scheduled one to three times per week. Due to the potent effects of metolazone, it is essential to closely monitor body weight, electrolytes, and renal function.

### 5.4 Choice of Loop Diuretics in HF

Furosemide is the most prescribed loop diuretic worldwide for the management of HF due to its long history of use, greater physician familiarity, and lower cost. However, torsemide offers several potential advantages over furosemide, including more consistent absorption and longer duration of action [23]. Additionally, torsemide shows beneficial effects on the RAAS through its inhibition of aldosterone synthesis, secretion, and receptor binding [24]. These mechanisms may contribute to its capacity to reduce myocardial fibrosis, which is a key factor in the progression of HF [25, 26].

Clinical studies comparing torsemide and furosemide have yielded conflicting results. An earlier randomized, open-label study by Murray *et al*. [27] involving 234 HF patients suggested that torsemide might be more effective than furosemide in reducing hospital readmissions for HF and cardiovascular conditions. The TORIC open-label, non-randomized surveillance trial evaluated the safety and efficacy of torsemide in chronic HF. The trial confirmed the safety and tolerability of torsemide, and reported improved functional status and reduced mortality compared to furosemide [28]. A more recent retrospective analysis of 1440 HF patients from the Polish registry of the ESC compared outcomes between matched cohorts treated with either torsemide or furosemide. This study reported that torsemide improved the NYHA class, reduced the composite endpoint for all-cause mortality, and decreased hospitalization for worsening HF [29].

In contrast, other non-randomized studies found no significant differences in 30-day outcomes or mortality between torsemide and furosemide, including the PROTECT trial [30] and the ASCEND-HF trial [31]. However, these studies were limited by the lack of randomization and imbalanced baseline characteristics, as the torsemide groups were smaller (13% in ASCEND and 16.5% in PROTECT) and had worse initial health status compared to the furosemide groups. TRANSFORM-HF was a randomized, open-label clinical trial conducted across 60 hospitals in the US that compared torsemide to furosemide in the management of HF. Although initially designed to enroll around 6000 patients, the trial was stopped after randomizing 2859 patients—as recommended by an independent data and safety monitoring board-having met its target goals. The TRANSFORM-HF study concluded that torsemide did not reduce all-cause mortality, hospitalizations, or patient symptoms compared to furosemide [32, 33, 34, 35].

Several meta-analyses have also compared cardiovascular outcomes between torsemide and furosemide [27, 28, 29, 30, 31, 32, 34, 36, 37, 38, 39, 40, 41, 42, 43]. As summarized in Table 3 (Ref. [27, 28, 29, 30, 31, 32, 34, 36, 37, 38, 39, 40, 41, 42, 43, 44]), the findings from these studies have been inconsistent. Some, but not all meta-analyses indicated that torsemide was associated with improved NYHA functional status, better LVEF, and reduced HF hospitalization. While one meta-analysis showed a reduction in cardiovascular mortality [38], no other meta-analysis showed a statistically significant decrease in cardiovascular or all-cause mortality with torsemide.

**Table 3.** **Main published studies comparing torsemide and furosemide in heart failure: trials and meta-analyses**.

First Author/Trial		Year	Study design	Number of patients	NYHA class	Hospitalization: for HF	Hospitalization: all-cause	Mortality: CV	Mortality: all-cause
Trials									
	Murray [27]	2001	ROLT	234	Improved	Decreased	No change	Not reported	Not reported
	Cosín TORIC Study [28]	2002	NROLT^1^	1377	Improved	Not reported	Not reported	Not reported	Decreased
	Mentz PROTECT Trial [30]	2015	Non-Randomized	1004	Not reported	Increased	Not reported	Not reported	Increased
	Mentz ASCEND Trial [31]	2016	Non-Randomized	4177	Not reported	No change	No change	No change	No change
	Ozierański [29]	2019	Retrospective	1440	Improved	Decreased^4^	Not reported	Not reported	No change
	Mentz TRANSFORM Trial [32]	2023	ROLT	2859	No change	No change	No change	No change	No change
	Krim TRANSFORM Trial [34]	2024	ROLT^2^	838	No change	No change	No change	No change	No change
Meta-analyses									
	DiNicolantonio [36]	2012	Meta-analysis	471	Improved	Decreased	Not reported	No change	No change^7^
	Kido [37]	2019	Meta-analysis	5	Improved	No change	No change	No change	No change
	Abraham [38]	2020	Meta-analysis	19,280	Improved	Decreased^5^	No change	Decreased	No change
	Miles [39]	2019	Meta-analysis	8127	Improved	Decreased	Not reported	No change	No change
	Sherif [40]	2020	Meta-analysis	1598	Not reported^3^	No change^6^	No change	No change	No change
	Eid [44]	2021	Meta-analysis	10,740	Not reported	No change	No change	No change	No change
	Siddiqi [41]	2023	Meta-analysis	11,966	Not reported	Decreased	Decreased	No change	No change
	Singh [42]	2023	Meta-analysis	4127	Not reported	Decreased	Decreased	No change	No change
	Teixeira [43]	2024	Meta-analysis	4115	No change^3^	Decreased	No change	Not reported	No change

This table summarizes published comparisons of torsemide and furosemide in HF patients. Both randomized and retrospective study designs are included, reflecting both agreement and differences in reported clinical outcomes across studies. CV, cardiovascular; NYHA, New York Heart Association; ROLT, randomized open label trial; NROLT, nonrandomized open label trial; LVEF, left ventricular ejection fraction. ^1^ Patients treated with torsemide had features of greater disease severity. ^2^ This study is a subset of the TRANSFORM TRIAL on *de-novo* versus chronic HF, with 838 *de-novo* HF patients. ^3^ This study reported improvement in LVEF with torsemide compared with furosemide. ^4^ This was a composite end point of all-cause death or hospitalization for worsening HF. ^5^ Numerically lower risk of hospitalization for HF with torsemide, but borderline statistical significance (95% CI: 0.51–1.03, *p* = 0.07). ^6^ Torsemide was associated with a shorter hospital stay. ^7^ A 14% reduction in all-cause mortality was observed with torsemide, but this did not reach statistical significance.

An interesting recent development is the advent of subcutaneous furosemide injections that could be used to treat volume overload in HF patients in the outpatient setting. This is currently marketed as single-use, prefilled syringes with 80 mg of furosemide (Furoscix by scPharmaceuticals, MA, USA) that are infused by an automated injector syringe over 4 hours. These injections have been shown to result in therapeutic plasma furosemide levels within 30 minutes of initiating the injection, providing equivalent diuresis to 80 mg of IV furosemide given over a similar period [45, 46]. Although it could be argued that oral torsemide or bumetanide might achieve similar effects due to better bioavailability than oral furosemide, so far there have been no direct comparisons between these agents and subcutaneous furosemide. Nevertheless, subcutaneous furosemide is a step in the right direction and could be particularly useful in settings where enteric intake is limited by gastrointestinal disease.

### 5.5 Choice of Thiazide Diuretics in HF

Several studies have investigated whether chlorthalidone offers advantages over HCTZ due to its longer half-life and greater potency. In a meta-analysis of hypertension studies involving approximately 10,000 patients, chlorthalidone was associated with a greater reduction in blood pressure compared to HCTZ [47]. Additionally, a randomized controlled crossover trial compared chlorthalidone at 12.5 mg/day (titrated to 25 mg/day) with HCTZ at 25 mg/day (titrated to 50 mg/day) over 8 weeks in untreated hypertensive patients. Chlorthalidone was found to be more effective at lowering systolic blood pressure, as measured by 24-h ambulatory monitoring [48]. However, these differences were not observed with office blood pressure measurements.

The key question is whether the superior blood pressure control shown by chlorthalidone translates into improved outcomes for HF patients. A large cohort study involving 730,255 individuals from multiple databases found no significant improvement in cardiovascular outcomes with chlorthalidone compared to HCTZ [49]. Notably, the use of chlorthalidone was associated with a higher risk of renal and electrolyte disturbances. Similar findings were observed in a retrospective study that evaluated the clinical outcomes and safety of chlorthalidone versus HCTZ in 12,722 adults with varying levels of renal function [50]. Further evidence was obtained from a randomized controlled pragmatic trial involving 13,523 patients. This found no significant differences in cardiovascular outcomes, renal outcomes, or non-cancer mortality between chlorthalidone and HCTZ. However, the chlorthalidone group experienced a higher incidence of adverse events and hypokalemia [51].

Overall, these studies do not provide sufficient evidence in favor of chlorthalidone over HCTZ as a first-line antihypertensive agent. However, a noteworthy finding from a subgroup analysis of the randomized trial indicated that chlorthalidone may be associated with reduced cardiovascular events in patients with a history of myocardial infarction or stroke. Therefore, individualized use of chlorthalidone may be considered in patients who require more potent diuresis when HCTZ proves insufficient, as well as in those with a prior history of myocardial infarction or stroke and in whom the potential cardiovascular benefits could be more pronounced [51].

### 5.6 Establishing a Target Weight During Clinic Visits

When managing diuretic therapy, an obvious issue is whether the diuretic dose should be fixed between clinic visits, or whether the target weight should be maintained within a specific range. We argue that maintaining the patient’s weight within a target range between clinic visits is the optimal approach. This strategy empowers patients to adjust their diuretic dosage based on daily home weight monitoring.

**Fig. 2.**
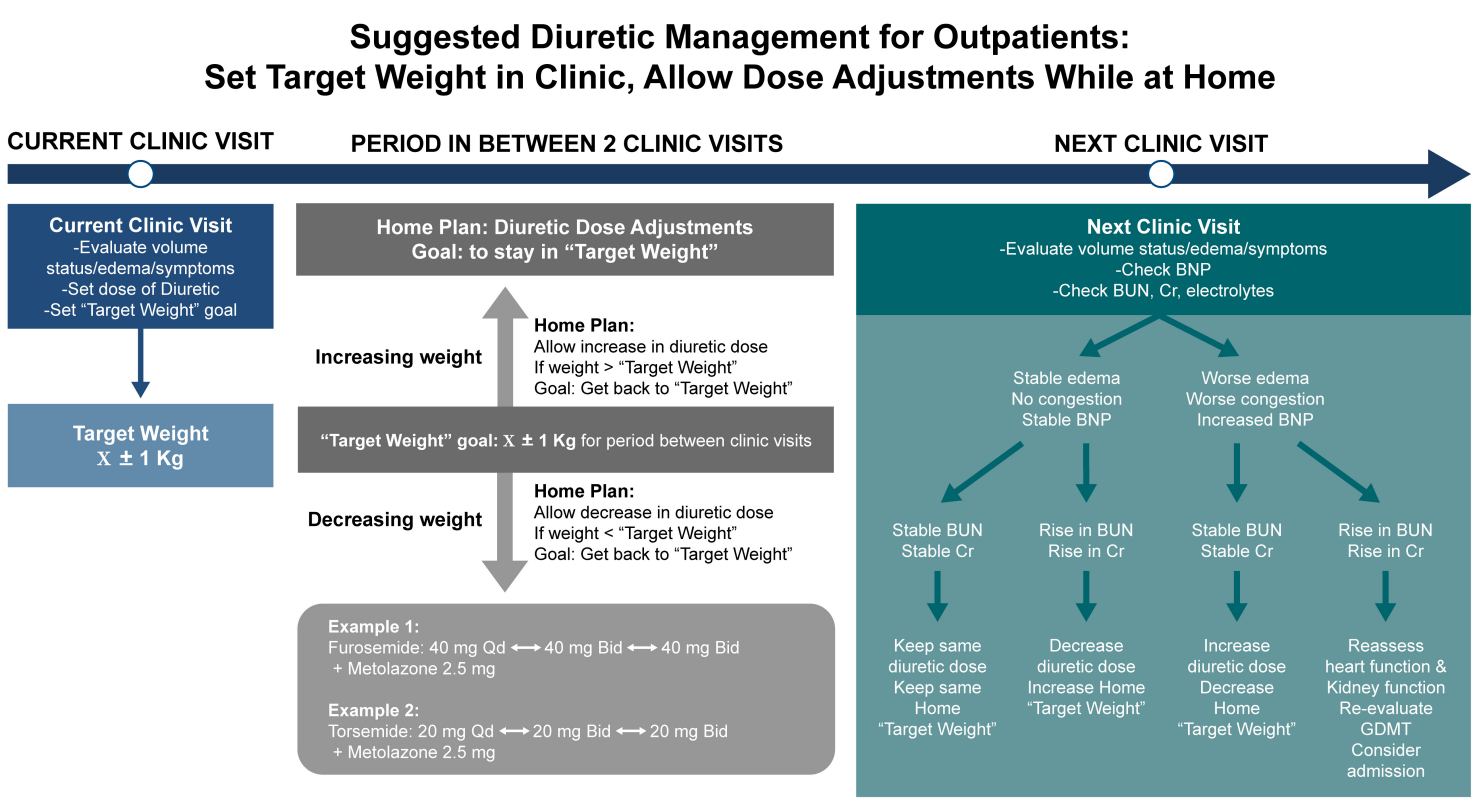
**Suggested diuretic strategy for outpatient diuretic management in patients with heart failure**. This original algorithm emphasizes setting an individualized target weight during clinic visits, followed by structured diuretic dose adjustments between visits based on deviations from the target. The figure illustrates clinical decision points for increasing or decreasing diuretic doses at home in response to changes in weight. BUN, blood urea nitrogen; BNP, brain natriuretic peptide; GDMT, guideline-directed medical therapy.

If the patient’s weight drops below the target range set at the last clinic visit, a reduction in diuretic dosage would be appropriate to avoid volume depletion and renal failure. Conversely, if the weight increases beyond the target range, the diuretic dose should be increased to avoid worsening congestion. Patients can make these adjustments using a physician-provided algorithm, as shown in Fig. 2. In the clinical judgment of this author, the strategy outlined in Fig. 2 helps to reduce the risks of cycling between HF exacerbations and prerenal failure between clinic visits, thus potentially decreasing hospitalization rates.

At each subsequent clinic follow-up visit, a comprehensive reassessment including electrolytes, kidney function, BNP levels, edema, symptoms, weight, and physical examination would guide the target weight range. To maximize the effectiveness of this approach, it is crucial to ensure timely follow-up visits for patients recently discharged from hospital, allowing for prompt adjustment of diuretic doses. This strategy promotes proactive outpatient management to maintain euvolemia and reduce the risk of decompensation or acute prerenal kidney failure.

### 5.7 Advanced Remote Monitoring Technologies for HF Patients

While the strategy outlined in Fig. 2 makes use of traditional methods to reduce hospitalizations for ADHF, remote monitoring technologies are revolutionizing HF management by enabling care beyond traditional clinical settings [52]. Such technologies use a minimally invasive, subcutaneous insertable cardiac monitor (ICM) and wearable sensors to track various critical physiological metrics such as impedance, respiratory rate with heart rate, variability and rhythm. These are used to create a validated HF diagnostic risk score that can identify patients at high risk of an impending worsening of HF [53]. Such remote monitoring data would be linked to actionable intervention by health care professionals. In the recent ALLEVIATE-HF phase 1 randomized trial, Kahwash *et al*. [54] demonstrated that such a personalized ICM-based intervention strategy, when activated by high-risk alerts, can be safely implemented and beneficial for patients.

### 5.8 Electrolyte and Renal Function Monitoring in the Outpatient Setting

Regular monitoring of fluid and electrolyte balance is essential, as diuretics can lead to imbalances such as hypokalemia, hyponatremia, hypomagnesemia, and metabolic alkalosis. These imbalances can exacerbate HF symptoms and lead to additional complications that require hospitalization. Kidney function should also be closely monitored through serum creatinine and blood urea nitrogen (BUN) levels, as diuretics can reduce renal perfusion and potentially worsen kidney function.

A recent case evaluated by our nephrology consultation service in a tertiary care hospital illustrates the severe adverse effects that occurred in a patient following the potentiation of diuresis. The case was a 70-year-old male with morbid obesity, CKD stage 3, hypertension, HFrEF, and lower extremity edema. He had been receiving chronic furosemide therapy at 40 mg twice daily, and presented to the emergency department (ED) with a three-week history of generalized weakness, gait abnormalities, dizziness, and frequent falls. The patient reported that around the time these symptoms began, he had visited his usual outpatient clinic, where metolazone 5 mg PO daily was added to his diuretic regimen due to worsening lower extremity edema and orthopnea. While this adjustment led to significant improvement in his edema, it coincided with the onset of his other symptoms. Upon presentation to the ED, he was found to have acute renal failure, along with critical hypokalemia and metabolic alkalosis. The critical electrolyte derangements in this patient are summarized in Table 4. He was managed with prompt and repeated IV KCl infusions for profound hypokalemia, and cautious IV saline administration for prerenal azotemia. The severe metabolic alkalosis was treated conservatively. Four hospital days were required to replenish the severe potassium deficit and resolve the prerenal kidney failure, and six days to correct the metabolic alkalosis. This case highlights the critical importance of outpatient monitoring of renal function and electrolytes following an escalation in diuretic therapy.

**Table 4.** **Electrolyte and renal complications following outpatient diuretic escalation in a heart failure patient**.

Date	Day -34	Day 0	Day 2	Day 4	Day 6	Day 9
Sodium (mEq/L)	142	131	138	138	136	135
Potassium (mEq/L)	3.8	2.1	2.3	3.5	3.1	3.9
Chloride (mEq/L)	101	69	79	91	94	98
Carbon Dioxide (mEq/L)	31	49	46	36	29	25
Anion gap (mEq/L)	10	13	13	11	13	12
Blood urea nitrogen (mg/dL)	27	106	66	41	40	38
Creatinine (mg/dL)	1.43	2.27	1.61	1.70	1.48	1.65
Calcium (mg/dL)	10.0	10.8	10.1	9.9	10.0	10.0
Magnesium (mg/dL)	-	3.2	2.7	2.4	2.2	2.2
Phosphorous (mg/dL)	-	2.2	2.2	2.6	3.1	3.0

This table highlights the case of the potential adverse effects of diuretic therapy, including hyponatremia, hypochloremia, hypokalemia, metabolic alkalosis, and acute kidney injury (AKI), which culminated in hospitalization of the patient. During the patient’s last outpatient clinic visit on day -34, laboratory data were collected and these are compared with those at presentation in the emergency department (ED) (Day 0). Presentation to the ED was triggered by confusion, frequent falls, AKI, and severe electrolyte disturbances. The response was conservative management, including cautious fluid resuscitation. A gradual improvement in both electrolyte abnormalities, acid-base status and azotemia was subsequently documented.

Through careful monitoring and timely diuretic dose adjustments, diuretics can effectively manage symptoms while minimizing adverse effects in outpatients with HF. If there is worsening of congestive symptoms, an increase in the diuretic dose should be considered. However, if there is a notable decline in kidney function in otherwise clinically stable patients, as evidenced by rising creatinine or BUN levels, the dosage of diuretics may need to be adjusted to prevent further renal impairment. This often involves reducing the diuretic dose, or switching to a less potent diuretic to maintain a delicate balance between managing fluid overload and preserving renal function. Patient education and inclusion with regards to recognizing the signs of fluid imbalance (shown in Fig. 2), and attendance at regular follow-up appointments are critical for the optimal management of HF with diuretic therapy.

## 6. Use of Diuretics for Acute Decompensated Heart Failure Patients

**Fig. 3.**
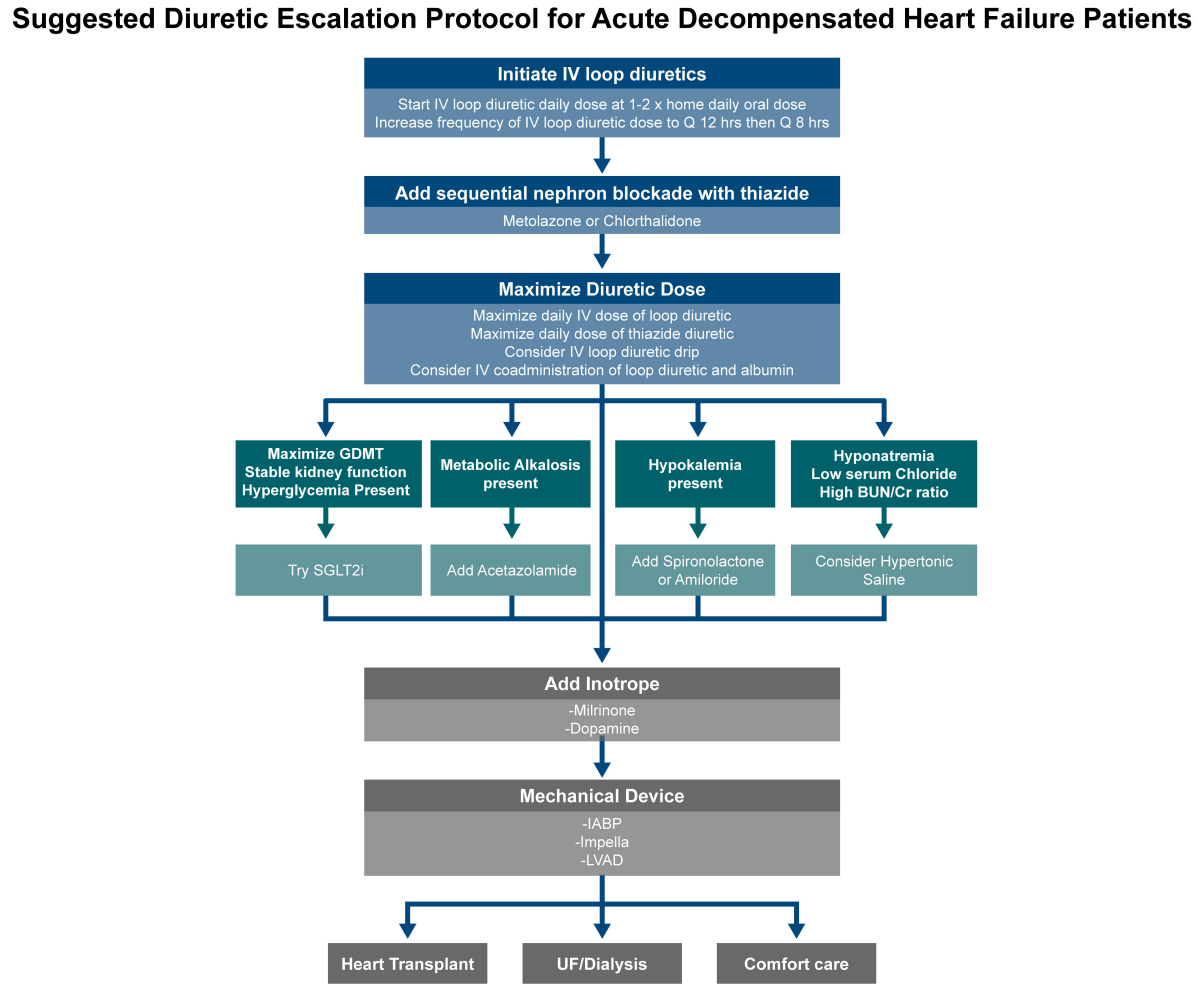
**Stepwise diuretic escalation protocol for patients with acute decompensated HF (ADHF)**. This schematic outlines a practical approach for the escalation of diuretic therapy in the inpatient setting, including initiation of IV loop diuretics, dose adjustments, and the addition of adjunctive agents in cases of insufficient decongestion. This protocol emphasizes a structured strategy to support timely volume management and optimize patient outcomes. SGLT2i, sodium glucose transporter 2 inhibitors; IABP, intra-aortic balloon pump; LVAD, left ventricular assist device; UF, ultrafiltration.

Patients with more advanced HF tend to decompensate and require hospitalization or treatment in a dedicated outpatient ambulatory outpatient HF center. IV diuretics would then be given to manage symptomatic volume overload and central vascular congestion. Worsening congestion in HF is a predictor of mortality, and IV administration of loop diuretics such as furosemide is the mainstay of decongestive treatments in hospitalized patients with advanced or decompensated HF. Under physiological conditions, IV injection of loop diuretics acts promptly on the ascending limb of the loop of Henle, inhibiting sodium and chloride reabsorption, and leading to enhanced urinary sodium excretion. Successful decongestion relieves symptoms of HF, shortens the hospital stay, and improves 30-day post-hospitalization clinical outcomes in patients with HF [55]. Fig. 3 outlines the diuretic escalation protocol for patients with ADHF.

### 6.1 Resistance to Loop Diuretics in HF

Resistance to loop diuretics in HF is well established [6] and driven by several well-characterized pathophysiological and compensatory mechanisms:

(1) Neurohumoral activation [20, 21]: The cascade of events is induced by an increase in venous congestion, with elevated CVP and PCWP. This congestion triggers RAAS activation. It also leads to heightened sympathetic nervous system activity and increased secretion of vasopressin and atrial natriuretic peptide. Together, neurohumoral changes increase renal vascular resistance. reduce renal blood flow, and cause a fall in GFR. Consequently, salt and water excretion is diminished [20, 21], ultimately contributing to diuretic resistance [56].

(2) Venous congestion: This leads to a rise in central venous pressure that is transmitted to the renal veins.

(3) Renal tubular resistance: The renal tubular response to loop diuretic was found to be blunted following chronic diuretic use [57]. This may be due to the increased number of thiazide-sensitive NaCl cotransporters and transport activity in the distal convoluted tubule (DCT) with the prolonged use of loop diuretics [8].

### 6.2 Natriuresis Following IV Loop Diuretics

The effectiveness of loop diuretics is typically assessed by measuring urine volume and sodium excretion. An adequate natriuretic response is indicated by a urine sodium concentration >50–70 mmol/L within 2–4 hours after diuretic administration [58]. However, the natriuretic effect of a single dose of loop diuretics is generally short-lived, lasting about 4–6 hours. This short duration may necessitate frequent dosing or continuous infusion to maintain therapeutic efficacy. Monitoring urine sodium levels and volume is crucial for tailoring diuretic therapy. For example, a urine sodium concentration <50 mmol/L may indicate diuretic resistance or insufficient dosing. In such cases, increasing the loop diuretic dose or adding a diuretic from another class, such as a thiazide, may help to overcome resistance and enhance natriuresis. Notably, the Enact-HF study demonstrated that protocol-guided monitoring of natriuresis led to greater cumulative diuresis within the first 48 hours of hospitalization, contributing to faster decongestion. This approach was suggested to allow clinicians to adjust diuretic therapy to achieve the desired natriuretic effect [58, 59]. Therefore, regular assessment of urine sodium and volume is essential for optimizing diuretic therapy in hospitalized patients and achieving faster decongestion.

### 6.3 Continuous Infusion or Intermittent Dosing of Loop Diuretics

The administration of loop diuretics, such as furosemide, can be performed either through intermittent IV dosing or continuous IV infusion. Intermittent IV dosing involves administering the diuretic at set intervals, often resulting in a peak-and-trough effect in drug concentration that can lead to periods of suboptimal natriuresis between doses. In contrast, continuous IV infusion maintains a steady plasma concentration of the diuretic, resulting in a more consistent natriuretic response. This continuous exposure can enhance sodium excretion and diuresis, potentially reducing the risk of rebound sodium retention that may occur with intermittent dosing. Several studies have compared the two administration methods, but the results have been conflicting. For example, a prospective, double blind randomized trial comprising 308 patients with ADHF found no significant difference in outcome between bolus versus continuous infusion [60]. However, a single center, double-blind randomized trial on 80 patients with ADHF who were randomized to bolus versus continuous furosemide infusion found the latter was associated with better decongestion [61]. A recent meta-analysis of 8 randomized trials that compared continuous and bolus furosemide in ADHF found no difference between continuous infusion and bolus furosemide for all-cause mortality, length of hospital stays and electrolyte disturbances [62]. However, continuous infusion was superior to bolus administration regarding urine volume and reduction in BNP [62]. These findings suggest that continuous infusion may provide superior natriuretic efficiency for patients requiring aggressive diuresis, but not in terms of the overall clinical outcomes. Therefore, the choice between the two methods of administration should be individualized, taking into consideration factors such as patient response, underlying conditions, and logistical factors in the healthcare setting.

### 6.4 Loop Diuretics and Albumin Co-Administration

The coadministration of furosemide with albumin has been a subject of much controversy [63, 64, 65, 66]. Albumin binding of furosemide is essential for its secretion into the renal tubule where it exerts its therapeutic effects [67]. In a recent observational study on albumin binding capacity, a higher albumin binding capacity was associated with increased free furosemide in the urine and was positively correlated with increased urine excretion.

A meta-analysis on the co-administration of albumin and loop diuretics in hypoalbuminemic patients with diuretic resistance included 10 studies, of which 8 had a crossover design. This analysis found only transient benefits at 8 hours, with the benefits no longer present at 24 hours [65]. A more recent meta-analysis [63] compared the effect of furosemide and albumin co-administration versus furosemide alone in 13 prospective studies with 422 participants that met the inclusion criteria [63]. The analysis revealed that furosemide with albumin co-administration led to more pronounced diuresis than furosemide alone. This augmented diuresis effect was more pronounced when the baseline serum albumin level was <2.5 g/dL, and in participants with reduced renal function. The authors concluded that the combination of furosemide with albumin may provide clinical benefits in patients with diuretic resistance and in whom diuretic doses have been maximized, and in patients with severe hypoalbuminemia. However, all investigators have acknowledged the high variability in responses and called for larger scale randomized controlled trials (RCTs) to further examine the potential role of this strategy [63, 65].

### 6.5 Combination Diuretics in ADHF: Loop Diuretic With Metolazone or Chlorothiazide

The treatment of decompensated CHF requires prompt diuresis. Loop diuretics are the mainstay of treatment through their ability to act on the ascending loop of Henle to inhibit sodium and chloride reabsorption, resulting in significant diuresis. However, in advanced CHF the efficacy of loop diuretics alone can diminish due to compensatory mechanisms and undesirable changes in renal function. The use of combination diuretic therapy to achieve sequential nephron blockade becomes essential in managing patients that exhibit resistance to loop diuretics. This approach involves using loop diuretics alongside thiazide-like diuretics to achieve synergistic effects, enhancing fluid removal and alleviating symptoms of fluid overload. Metolazone is often combined with loop diuretics to overcome this resistance. The action of metolazone on the distal convoluted tubule provides a complementary mechanism that enhances sodium and water excretion, thus making it effective in cases where loop diuretics alone are insufficient. Since it can be administered intravenously, chlorothiazide offers a robust diuretic response in acute settings when used in combination with loop diuretics. These thiazide-loop diuretic combinations are particularly valuable in managing refractory edema in advanced CHF. Modest worsening of serum creatinine has been observed with the use of metolazone in certain studies. Hence, careful monitoring of electrolytes, renal function, and overall fluid balance is essential to prevent adverse effects such as hypokalemia, hyponatremia, and worsening renal function (Table 2).

### 6.6 Combination Diuretics in ADHF: Loop Diuretic With Metolazone Versus Acetazolamide

Historically, acetazolamide and other older carbonic anhydrase inhibitors have been of little or no benefit in managing acute CHF [68, 69, 70]. Their natriuretic effects were transient, and induction of acidosis further limited their chronic efficacy [69]. However, renewed interest in the use of acetazolamide in combination with loop diuretics has emerged following the ADVOR trial. This RCT included hospitalized patients with ADHF, BNP (specifically the N-terminal proBNP) >1000, GFR >20 mL/min/1.73 m^2^, and who were not receiving SGLT2i. They were given bumetanide at twice their home dose and were randomized to acetazolamide 500 mg daily or placebo. The primary outcome was decongestion, assessed within three days of admission using a congestion score, without the need for diuretic escalation [71]. The acetazolamide group achieved the primary endpoint, with decongestion observed in 42.2% of patients compared to 30.5% in the placebo group (*p* < 0.001). Additionally, the acetazolamide group had better congestion scores at discharge (78% vs. 62.5%). While cumulative diuresis and natriuresis were statistically significant in favor of acetazolamide, the absolute differences were modest, with about 0.5 liters of additional urine output. A *post-hoc* analysis of the ADVOR trial concluded that acetazolamide is more effective in patients with higher baseline CO_2_ levels (≥27 mmol/L) [72], suggesting its use in patients with metabolic alkalosis.

Whereas the ADVOR study compared acetazolamide to placebo on a background of loop diuretics, Abbo *et al*. [73] evaluated acetazolamide versus metolazone as an add-on to loop diuretics in a group of patients with decompensated HF who were already receiving GDMT. The patients in this study were ambulatory, but received high doses of IV furosemide in a dedicated outpatient center. The results favored metolazone [73], which showed greater weight loss, natriuresis and urine volume compared with acetazolamide. Furthermore, the diuresis achieved with acetazolamide in the ADVOR trial did not exceed that achieved by standard escalation protocols in the Bart Trial [74]. Although there was a slight worsening of renal function associated with metolazone use, this did not have a significant impact on the rate of hospitalization.

### 6.7 Use of Hypertonic Saline in ADHF

It is common and conventional practice to restrict salt intake in patients with HF. Undoubtedly, an increase in salt intake would be harmful to some patients with HF by driving fluid intake and congestion. Moreover, the main purpose of diuretics is to remove salt and water from the body to achieve decongestion. However, despite the widely established dogma that salt restriction is one of the mainstays of dietary management of HF, studies on salt restriction have failed to show favorable changes in weight [75] or a reduction in mortality [76]. Also, a reduction in dietary sodium to <100 mmol in an international, open-label RCT [77] of ambulatory HF patients did not reduce clinical events.

There remains concern that sodium restriction leads to activation of the RAAS [78], which is associated with untoward effects in HF. Low serum chloride is associated with worse diuretic responsiveness and increased mortality [79, 80, 81]. Given the lack of efficacy and potential for long term harm, the ESC guidelines have gradually relaxed sodium chloride restrictions in their latest consensus statement to no more than 5 grams per day in patients with HF [82].

Contrary to traditional clinical wisdom, several studies [83, 84, 85, 86, 87, 88, 89, 90, 91] have evaluated the benefit of IV hypertonic saline (or oral salt administration) in combination with loop diuretics in patients with HF. Several diverse hypothetical mechanisms have been proposed for the administration of a salt load to decongest patients with HF [92], including: (1) reduction in the deleterious effects of neurohumoral activation; (2) correction of hyponatremia and hypochloremia which are associated with increased mortality, since hypochloremia is associated with neurohumoral activation and diuretic resistance [79, 81]; (3) utilization of the hypertonic effect to draw fluids from the intracellular compartment and interstitial spaces, with the diuretics facilitating their excretion from the intravascular volume; and (4) overcoming diuretic resistance of the DCT in HF [56]. Interestingly, all these studies concluded that administration of a hypertonic saline solution (HSS) in ADHF resulted in improved congestive symptoms and renal outcomes [84, 85, 86, 87, 88, 89, 90, 91, 93]. The dose of HSS used in these studies was variable, ranging between 1.4% and 7.5% saline. A common dose was 150 mL of 3% HSS given over 30 minutes and coupled with a high dose of loop diuretic (>200 mL of furosemide).

A meta-analysis of 10 RCTs conducted up to June of 2022 included 3013 patients and showed that HSS plus furosemide significantly reduced the length of hospital stay, weight, creatinine, and BNP, while increasing urine output, serum sodium, and urine sodium. However, the meta-analysis also concluded that the improved surrogate outcomes reported in ADHF patients in these studies were of low to intermediate quality [92], and that adequately powered RCTs were still needed to evaluate possible benefits in HF readmission and mortality.

More recently, the Osprey-HF trial [94] evaluated oral NaCl tablets at 6 grams daily for four days in a double blind, placebo-controlled study in 65 patients from a single center. Although no difference was found between the two groups in terms of serum creatinine or change in weight at four days, the trial was underpowered. The Salt-HF trial also evaluated the effect of a modest, single infusion of HSS in 167 ambulatory patients with HF. This trial was a multicenter, randomized double-blind study in which a 1-hour infusion of IV furosemide with HSS was compared to furosemide alone. No improvements in short-term diuresis or congestion parameters were observed compared to furosemide alone. The authors acknowledged that the administered salt load in their study was very modest, and concluded that additional trials using higher salt concentrations, increased infusion frequency, or longer evaluation periods were needed [95, 96]. Thus, administration of HSS, or oral salt loading, continues to be controversial and should likely be reserved for patients with hyponatremia and hypochloremia, as a final attempt after failure of maximization of segmental nephron blockade [79].

### 6.8 SGLT2 Inhibitors in ADHF

SGLT2i are known to provide osmotic diuresis [97, 98]. They also provide modest natriuresis through complex mechanisms [99, 100, 101], including SGLT2 inhibition and blockade of luminal membrane sodium hydrogen exchanger 3. These effects can potentially contribute to decongestion and are likely to offer an adjunct to diuretic therapy in HF [99]. SGLT2i have been used in combination with loop diuretics in studies on acute HF. Their use has led to a reduction in the total dose of diuretics, as well as enhanced diuresis [102, 103].

Beyond diuresis, several large, randomized studies using various SGLT2i have shown improved cardiovascular outcomes when used in HF patients [104, 105, 106, 107, 108, 109]. The potential mechanisms underlying such benefits are varied, but may be due to a decrease in plasma volume, reduction in sympathetic system and renin angiotensin system activities, and the blunting of inflammatory mechanisms [110, 111]. Studies in experimental animal systems have led to an increased interest in the use of SGLT2i in HF, with observations including: (1) SGLT2 overexpression in the kidneys of experimental HF animals; (2) activation of the sympathetic system in HF, as well as a norepinephrine-induced increase in trafficking of SGLT2 to the proximal luminal membrane of renal tubular cells, leading to increased salt and water retention; (3) chronic inhibition of SGLT2 reduced renal sympathetic nerve activity and renal inflammation and increased urine flow and sodium excretion in rats with CHF; (4) SGLT2i reduced renal afferent and efferent nerve activity in animals with HF; and (5) other studies showed that SGLT2i reduced inflammatory markers in rats with HF [112]. These studies provide additional insight into the potential mechanisms by which the early use of SGLT2i in ADHF could achieve both diuretic efficacy and renal protective effects.

### 6.9 Rate of Diuresis in ADHF

Congestion is the predominant symptom of ADHF. In such instances, the relief of congestive symptoms is the most compelling need for acutely hospitalized patients. However, <25% of patients become congestion-free within three days after initial hospitalization [8, 113]. Prompt initiation of aggressive diuretic strategies in the ED leads to improvements in inpatient mortality, length of stay, symptom duration, and overall mortality [114]. It is recommended to initiate diuresis through IV administration of high-dose loop diuretics, starting at approximately 2–2.5 times the usual home dose. This should be followed by dose escalation, using guided natriuresis and diuresis to enhance decongestion [8].

Dose escalation is recommended for persistent symptoms of congestion if the urine volume is <3–5 liters per day, or if urine sodium is <50–70 mEq/L. Published algorithms recommend escalating the dose of loop diuretics, then combining them with escalating doses of thiazide diuretics to achieve a cumulative urine output of 6 liters within 48 hours [8, 113]. The choice between intermittent loop diuretic boluses or continuous infusion is not a decisive factor. Similarly, the selection of oral metolazone as opposed to IV chlorothiazide is not critical.

Additional options include adding acetazolamide in the context of coexisting alkalosis, or potassium-sparing diuretics in the presence of hypokalemia. Interestingly, HSS or SGLT2i may serve as adjunct therapies in certain scenarios. While rapid decongestion is generally well tolerated [59, 115], it is essential to monitor the patient’s hemodynamics, kidney function, and electrolytes to ensure safety. However, the achievement of long-term cardiac outcomes will ultimately require GDMT [99].

### 6.10 Monitoring Parameters in Hospitalized Patients With ADHF

The efficacy of diuresis in hospitalized patients with ADHF is judged by achieving urinary output goals, resolving physical signs of volume overload, and alleviating symptoms of decongestion. Safety is assessed by monitoring hemodynamics, electrolytes, and renal function. Monitoring of electrolytes is crucial for the replacement of diuretic-induced losses in potassium and magnesium. It is also essential to monitor serum sodium because hyponatremia is common in decompensated HF and often needs to be addressed. Stable renal function is critical for the success of diuresis and for patient outcomes. Most hospitalized patients exhibit a modest increase in creatinine with successful, large volume diuresis. This is expected and should not deter clinicians from proceeding with the main task of achieving optimal volume control. Studies have shown that if creatinine rises but congestion is relieved, the clinical outcomes are better than if baseline creatinine is maintained but the patient fails to decongest [116, 117]. Hence, a modest rise in creatinine should not be construed as a negative sign. However, if a progressive increase in creatinine occurs, a reduction in diuresis intensity would be necessary due to concerns about other causes of renal failure, including intrinsic kidney failure or the emergence of cardiorenal syndrome.

### 6.11 Diuretics and Acute Cardiorenal Syndrome

A progressive rise in creatinine and azotemia in the context of ADHF raises the possibility of cardiorenal syndrome (CRS). The pathophysiology of CRS includes worsening neurohumoral activation, vasoconstriction, a rise in venous pressure, and a decrease in kidney perfusion. This is followed by a vicious cycle of reduced renal plasma flow, reduced GFR, salt and fluid retention, and further congestion. CRS patients typically have persistent congestive symptoms and tend to exhibit hypotension and diuretic resistance. Lactic acidosis and liver test abnormalities are also frequently observed. The occurrence of CRS is an ominous sign and should prompt the initiation of inotropic agents such as Dobutamine or Milrinone. Optimization of heart function may lead to the reversal of CRS, but mechanical circulatory devices are often needed, such as intra-aortic balloon pump, percutaneous microaxial pumps, or LV assist devices. Unless acute tubular injury has already occurred, inotropic support or mechanical support can restore the effectiveness of diuretics in CRS. Diuretics therefore continue to be important agents in these instances - but with improved cardiac output their doses can be reduced.

### 6.12 Monitoring of Central Filling Pressures in ADHF

In states of ADHF, right heart catheterization (RHC) offers valuable information about the pulmonary artery (PA) pressure, PCWP, transpulmonary pressure gradiant (TPG), right atrial (RA) pressure, and right ventricular pressure. This information regarding central filling pressures and RA pressure helps to guide diuretic doses aimed at reducing central filling pressures.

High PA pressure may be due to high LV filling pressures (also called post-capillary pulmonary hypertension), intrinsic pulmonary arterial hypertension (also called pre-capillary pulmonary hypertension), or both (mixed pre- and post-capillary pulmonary hypertension). Several scenarios may be encountered and should be managed using different approaches. The corresponding RHC findings and management strategies for four patients with different underlying diseases are presented in Table 5. Predominantly post-capillary pulmonary hypertension requires continued diuresis to reduce LV filling pressures. However, predominantly pre-capillary primary pulmonary hypertension is best treated with pharmacologic agents to reduce pulmonary vascular constriction. RHC may also show mixed patterns of pre- and post-capillary pulmonary hypertension, with diuretics being useful in such cases. RHC is often used to guide diuresis intensity during the most advanced stages of ADHF, and is often repeated following intervention. It is also a reliable tool for monitoring a patient’s diuretic requirements post heart transplantation.

**Table 5.** **Right heart catheterization findings in illustrative cases of HF**.

Case	CI (L/min/m^2^)	MRAP (mmHg)	MPAP (mmHg)	PCWP (mmHg)	TPG (mmHg)
1	1.34	18	48	24	24
2	1.96	24	52	36	16
3	3.46	23	57	20	37
4	3.07	26	36	30	6

Four illustrative cases of HF are shown. This table presents key hemodynamic measurements obtained via right heart catheterization (RHC) that help to characterize HF pathophysiology and guide individualized management strategies in diverse clinical scenarios. CI, Cardiac Index; MRAP, mean right atrial pressure; MPAP, mean pulmonary artery pressure; PCWP, pulmonary capillary wedge pressure; TPG, transpulmonary gradient; ADHF, acute decompensated HF.

Case 1 is a patient with non-ischemic cardiogenic shock with mixed pre- and post-capillary pulmonary hypertension. Diuretics were used to reduce central filling pressures and control volume overload. The patient eventually received an LVAD. Case 2 is a patient with cardiac aTTR amyloidosis with predominantly post-capillary pulmonary hypertension. Diuretics were used to reduce central filling pressures and control volume overload. The patient eventually received a heart transplant. Case 3 is representative of patients with chronic thromboembolic pulmonary hypertension (CTEPH), with predominantly severe pre-capillary pulmonary hypertension. This patient received diuretics to manage associated high PCWP, but the mainstay of treatment was the use of pharmacologic agents to reduce pulmonary hypertension. The patient eventually underwent pulmonary thromboendarterectomy. Case 4 is a patient with a 6-year-old heart transplant admitted with ADHF. He had elevated PCWP due to acute decompensation of the LV, but had normal TPG. He was initially treated with diuretics to achieve decongestion, then had an endomyocardial biopsy that showed no rejection of the allograft. He was then referred to interventional cardiology to perform left heart catheterization.

Another promising tool to monitor LV filling pressures is a sensor implanted in the PA to measure PA pressures as a surrogate for LV filling pressures. This enables early intervention, such as an increased dose of diuretics in response to elevated PA pressure, thereby preventing hospitalization for exacerbation of HF. Such a PA device has shown convincing clinical results in terms of its efficacy and safety for the remote monitoring of patients with HF [118, 119, 120].

## 7. Conclusion

Diuretics play an essential role in the management of HF, serving as the primary agents for relieving the symptoms of fluid overload in both outpatient and inpatient settings. The selection and dosing of diuretics should be guided by the degree of congestion, stage of HF, prior diuretic response, and comorbid conditions such as CKD.

After initiation, the diuretic regimen must be dynamically adjusted according to the patient’s natriuretic response and the degree of decongestion achieved. Diuretic resistance is a common clinical barrier, often resulting from neurohormonal activation and compensatory sodium reabsorption in distal segments of the nephron. In such cases, sequential nephron blockade using combinations of loop diuretics with thiazide-type or carbonic anhydrase inhibitors increase diuretic efficacy and facilitate volume removal.

Careful follow-up of HF patients in the outpatient setting is critical. Clinical assessment and regular monitoring of weight, renal function, and electrolytes help to guide dose adjustments and allow early intervention before significant decompensation occurs. Establishing a “target weight” and encouraging patient engagement in tracking symptoms and weight trends can empower self-management and reduce the risk of hospitalization.

For hospitalized patients with ADHF, timely and effective volume reduction is essential. The measurement of urine sodium concentration within hours of diuretic administration is emerging as a practical tool to assess diuretic responsiveness and guide escalation. When coupled with vigilant monitoring, large-volume diuresis has proven to be both safe and effective in accelerating clinical recovery and reducing the length of hospital stay.

Importantly, while diuretics are indispensable for symptom control and decongestion, they are not substitutes for disease-modifying therapies. GDMT, including SGLT2i, RAAS blockers, beta-blockers and mineralocorticoid receptor antagonists, remain the cornerstone of long-term HF management and must be optimized in parallel with diuretic use.
